# World Checklist of Opiliones species (Arachnida). Part 1: Laniatores – Travunioidea and Triaenonychoidea

**DOI:** 10.3897/BDJ.2.e4094

**Published:** 2014-11-05

**Authors:** Adriano B. Kury, Amanda Cruz Mendes, Daniele R. Souza

**Affiliations:** † Museu Nacional/UFRJ, Rio de Janeiro, Rio de Janeiro, Brazil; ‡ Universidade do Estado do Rio de Janeiro, Rio de Janeiro, Brazil; § Museu Nacional, Universidade Federal do Rio de Janeiro, Rio de Janeiro, Brazil

**Keywords:** Harvestmen, Afrotropical, Australasian, Nearctic, Neotropical, Palearctic

## Abstract

Comprising more than 6500 species, Opiliones is the third most diverse order of Arachnida, after the megadiverse Acari and Araneae. The database referred here is part 1 of 12 of a project containing an intended worldwide checklist of species and subspecies of Opiliones as Darwin Core archives, and it includes the superfamilies Travunioidea and Triaenonychoidea. These two superfamilies are often treated together under the denomination of Insidiatores. In this Part 1, a total of 571 species and subspecies are listed. Briggsidae and Cladonychiidae are both downgraded to subfamilies of Travuniidae. *Peltonychia* Roewer, 1935 is an available name and senior synonym of *Hadziana* Roewer, 1935 and is herein revalidated. Seven genera of Triaenonychidae described by Lawrence between 1931 and 1933 originally failed to comply ICZN rules for availability (Art. 13.3). All of them only became available when Staręga (1992) designated a type species for each. Therefore, the correct authorships of *Austromontia* Lawrence, 1931, *Biacumontia* Lawrence, 1931, *Graemontia* Lawrence, 1931, *Larifugella* Lawrence, 1933, *Mensamontia* Lawrence, 1931, *Monomontia* Lawrence, 1931 and *Rostromontia* Lawrence, 1931 are all Staręga, 1992. *Fumontana* Shear, 1977, originally referred only to subfamily Triaenonychinae (as opposed to Soerensenellinae then and not corresponding to present Triaenonychinae), not to any tribe (which in turn correspond to modern subfamilies) is herein included in the subfamily Triaenonychinae. *Picunchenops* Maury, 1988 originally not included in any tribe of Triaenonychidae, is herein included in the subfamily Triaenonychinae. *Trojanella* Karaman, 2005, originally ranked as Travunioidea incertae sedis, is herein included in the Travuniidae Travuniinae. *Nuncia ovata* Roewer, 1915 (synonymized with *Triaenonyx cockayni* Hogg, 1920 by Forster (1954), but with inverted precedence) is here combined as *Nuncia coriacea ovata* Roewer, 1915 as correct senior synonym instead of *Nuncia coriacea cockayni* (Hogg, 1920), which is current in the literature. *Neonuncia enderbei* (Hogg, 1909) is reaffirmed as the correct spelling for the species, while the deliberate change to *Neonuncia enderbyi* by Forster (1954) is an incorrect subsequent spelling.

## Introduction

### Placement and composition

For decades, Travunioidea and Triaenonychoidea have been historically regarded as a single unit, called Travunioidea (e.g., Martens 1980). Kury (2003) resurrected the name Insidiatores Loman 1900 to collectively refer to them, but subsequent authors failed to recover the monophyly of Insidiatores (Giribet and Kury 2007) and even of the component families (Sharma and Giribet 2011).

Both morphological (Mendes 2009) and molecular (Giribet et al. 2010,  Sharma and Giribet 2011) phylogenetic analyses found that the Palearctic “Triaenonychidae” group with expanded Travuniidae and Travunioidea. Kury (2013)  following results of Mendes (2009) fused Briggsidae and Cladonychiidae with Travuniidae, which is also supported by Giribet et al. (2010) and Sharma and Giribet (2011). Herein we adopt this scheme of classification, and downgrade Briggsidae and Cladonychiidae to subfamilies of Travuniidae further. Mendes (2009) found that the Triaenonychoidea *sensu stricto* are sister to Grassatores, not to Travunioidea, in contrast with Giribet and collaborators, who found Triaenonychidae as sister group of Travunioidea. Giribet et al. 2010 and Sharma and Giribet 2011 found Synthetonychiidae as the sister group to all other Laniatores, but this placement is not incorporated here.

The North American monotypic *Fumontana* Shear, 1977 is probably closely related to the southern triaenonychids, although its exact positioning in the family is still unknown (Giribet and Kury 2007, Thomas and Hedin 2008, Mendes 2009). It is currently the only representative of Triaenonychoidea from the Northern Hemisphere. The only analysis with broader representation of Insidiatores (Mendes 2009) found *Fumontana* closer to *Triaenonyx* Sørensen, 1886 than to *Soerensenella* Pocock, 1902, *Triaenobunus* Sørensen, 1886 and *Adaeum* Karsch, 1880 hence we consider this species here as a member of the subfamily Triaenonychinae.

According to the results of Mendes (2009), *Trojanella serbica* Karaman, 2005, currently considered a Travunioidea incertae sedis (Karaman 2005), groups with the European travunioids, forming a clade with *Travunia* Absolon, 1920, and based on those results we newly assign this species to Travuniidae, subfamily Travuniinae. Mendes also included *Picunchenops spelaeus* Maury, 1988 in her analyses. Originally this species was placed by Maury (1988) in Triaenonychinae, but not to any of its tribes (which roughly corresponds to subfamilies). In her results, Mendes found that this species is closer to *Triaenonyx* and other triaenonychines, thus we consider herein this species as a Triaenonychidae, subfamily Triaenonychinae (or in Maury's terms, Triaenonychini).

We provide additional nomenclatural considerations in the section "Additional information".

### Recent works on these groups

In the last years, some work has been done with North American Travunioidea. Shear (2008) synonymized a species of Cladonychiidae, *Phalangomma virginicum* Roewer, 1949 (originally assigned to Phalangodidae) with the travuniid *Erebomaster weyerensis* (Packard, 1888). Shear and Derkarabetian (2008) redefined Paranonychinae based on characters of the penis and proposed the synonymy of Kaolinonychinae as its junior synonym.  Derkarabetian et al. (2011) and Derkarabetian and Hedin (2014) have been studying the sclerobunines using modern integrative taxonomy to detect morphological homogeneous undescribed species, synonymies and the evolution of troglomorphisms (Derkarabetian et al. 2011, Derkarabetian and Hedin 2014). Most recent published work on Triaenonychoidea is only cursory and on Afrotropical taxa (e.g., Mendes and Kury 2012), while Australasian and Neotropical members are neglected of late.

## General description

### Purpose

This project is a checklist of all valid specific and subspecific names (counted together) of the arachnid order Opiliones. The project intends to deliver 12 parts for ease of handling and preparing manuscripts. This is part 1 of 12 and covers the two basal superfamilies of Laniatores – the Travunioidea and the Triaenonychoidea.

## Project description

### Title

World Checklist of Opiliones species (Arachnida).

### Personnel

Adriano B. Kury (Author, Content Provider, Metadata Provider), Amanda C. Mendes (Author, Content Provider), Daniele R. Souza (Author, Content Provider).

### Design description

This project aims to produce a general checklist of all the valid species and subspecies (which are counted together) names of harvestmen of the world (Arachnida, order Opiliones). That is, only senior homonyms and synonyms are included. Alternative unused combinations are not listed.

Given the bulk of the project, it is divided in 12 parts as follows (numbers of subsequent parts are subject to change):

Part 1. Laniatores – Travunioidea and Triaenonychoidea (571 spp)

Part 2. Laniatores – Grassatores incertae sedis, Samooidea and Zalmoxoidea (564 spp)

Part 3. Laniatores – Assamioidea (529 spp)

Part 4. Laniatores – Epedanoidea and Phalangodoidea (539 spp)

Part 5. Laniatores – lesser Gonyleptoidea (506 spp)

Part 6. Laniatores – Cosmetidae (729 spp)

Part 7. Laniatores – Gonyleptidae (760 spp)

Part 8. Cyphophthalmi and Dyspnoi (552 spp)

Part 9. Eupnoi – incertae sedis, Caddidae,  Neopilionidae and Phalangiidae (476 spp)

Part 10. Eupnoi – lesser Sclerosomatidae (289 spp)

Part 11. Eupnoi – Gagrellinae: Old World (741 spp)

Part 12. Eupnoi – Gagrellinae: New World (313 spp)

### Funding

This study has been supported by grants # 562149/2010-4 (PROTAX – OPESC project), # 504327/2012-7 (Sistema de Informações sobre a Biodiversidade Brasileira (SiB-Br) - Coleções Biológicas) and scholarship # 302116/2010-9 (PQ - AMMA project) from the Conselho Nacional de Desenvolvimento Científico e Tecnológico (CNPq).

## Geographic coverage

### Description

General spatial coverage: worldwide. The Travunioidea and Triaenonychoidea together have 307 Australasian species, 159 Afrotropical, 40 Nearctic, 38 Palearctic, 26 Neotropical and only 1 Holarctic. As Travunioidea is a typical Laurasian group, while Triaenonychoidea represents a mostly temperate Gondwana fauna, there are no records of these groups from Indo-Malaya. *Promecostethus unifalculatus* Enderlein, 1909 from Crozet Island, French Subantarctic Lands is here listed as Australasian.

## Taxonomic coverage

### Description

This part 1 of the checklist includes the Insidiatores Loman 1900, which represent the basal Laniatores. Superfamilies Travunioidea and Triaenonychoidea. The taxonomic outline for large groups of Opiliones used here follows Kury (2013).

**Figure 1.**
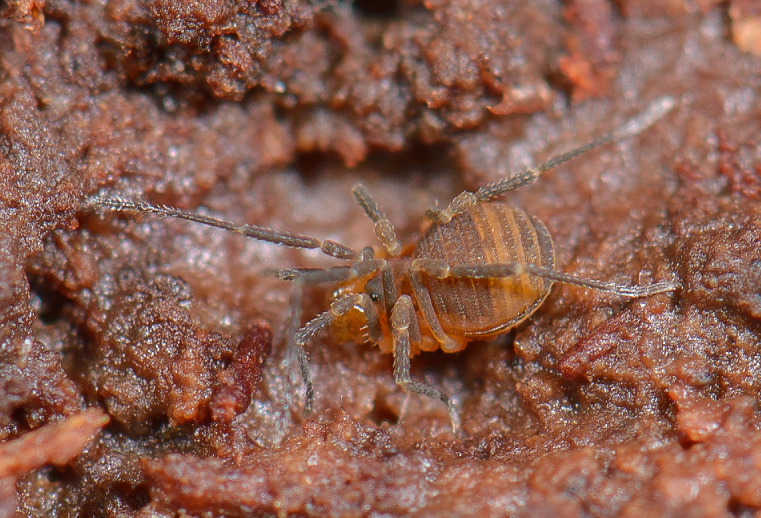
Paranonychidae, Paranonychinae, *Paranonychus brunneus* (Banks, 1893), adult, USA. Photo, ID and copyright © by Marshal Hedin. Image online at link.

**Figure 2.**
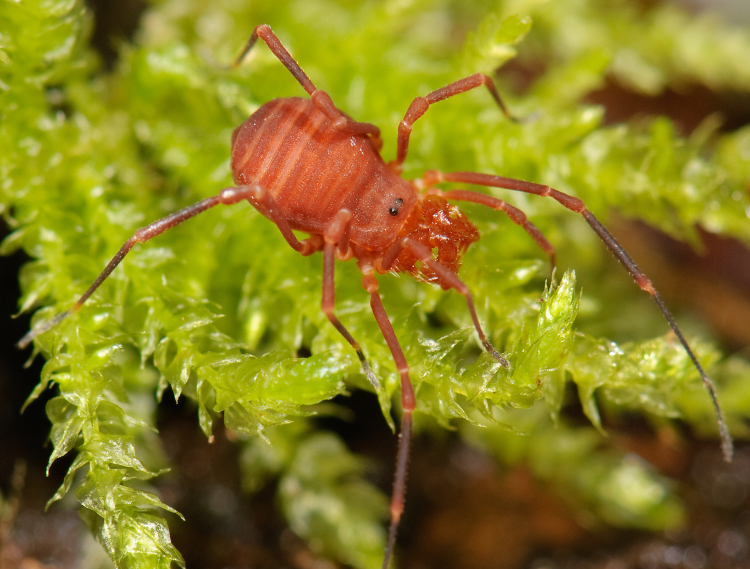
Paranonychidae, Sclerobuninae, *Sclerobunus nondimorphicus* Briggs, 1971, adult, USA, WA, Pacific Co.. Photo, ID and copyright © by Marshal Hedin. Image online at link.

**Figure 3.**
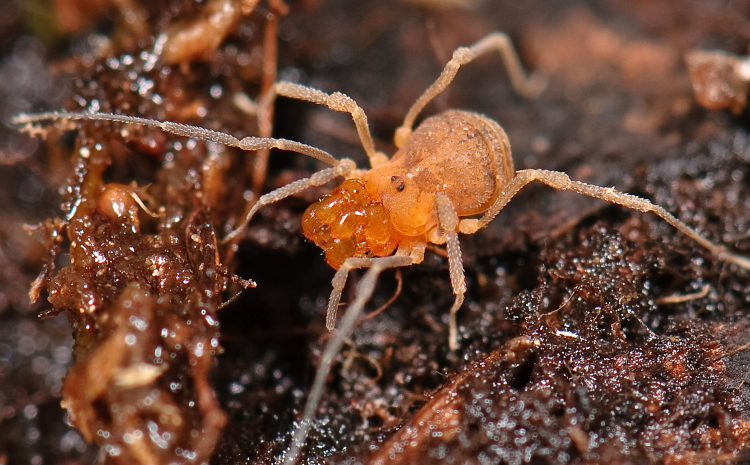
Travuniidae, Briggsinae,  *Briggsus* sp., adult, collected by C. Richart & S. Derkarabetian, 3 April, 2008, USA, OR, Clatsop Co. Photographed in lab. Photo,  ID and copyright © by Marshal Hedin. Image online at link.

**Figure 4.**
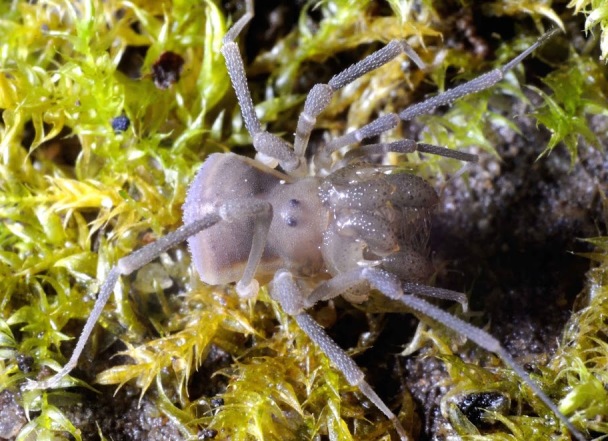
Travuniidae, Cladonychiinae, *Cryptomaster leviathan* Briggs, 1969, teneral adult, USA, OR, Curry Co., Lobster Creek Rd. Photo, ID and copyright © Axel Schönhofer. Image online at link.

**Figure 5.**
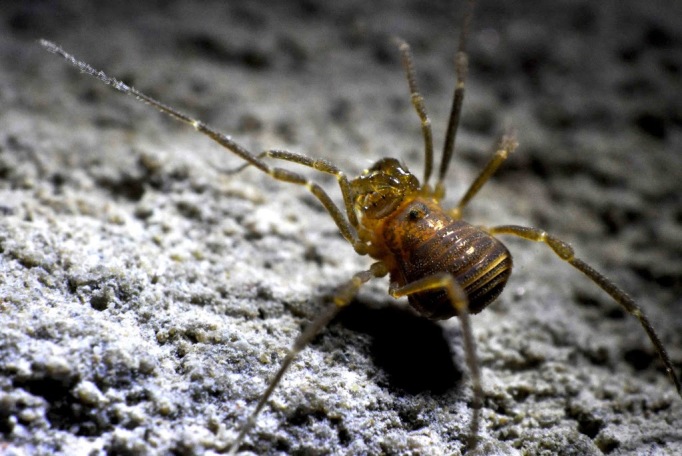
Travuniidae, Travuniinae, *Peltonychia leprieuri* (Lucas, 1861), adult, Italy Roncobello. Photo, ID and copyright © Axel Schönhofer. Image online at link.

**Figure 6.**
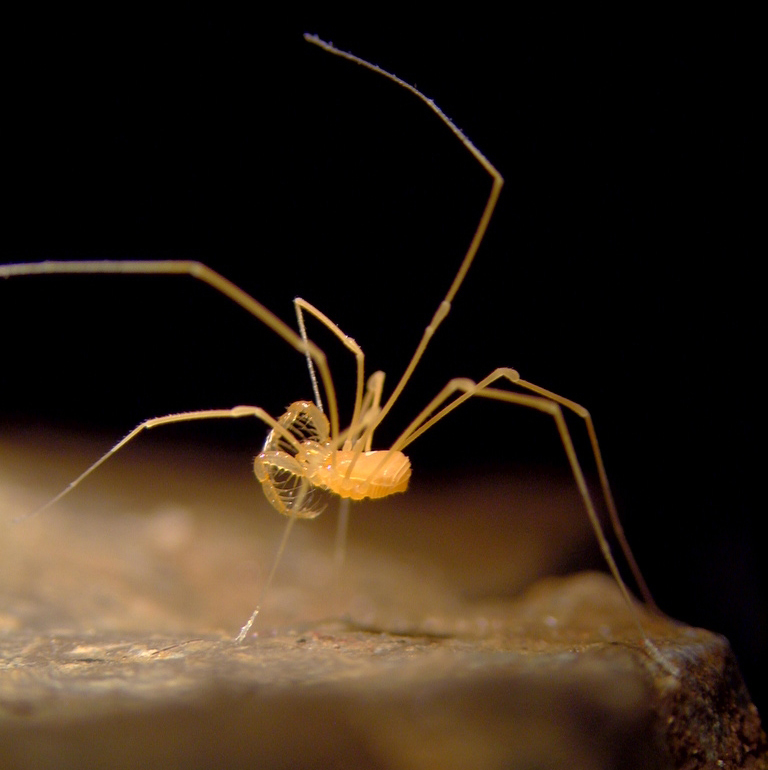
Travuniidae Travuniinae, *Trojanella serbica* Karaman, 2005, adult from Serbia, image cropped. Photo, ID and copyright © Ivo Karaman.

The Travunioidea have 78 valid species/subspecies, including 39 Nearctic, 38 Palearctic and 1 Holarctic taxa. Three families and 5 subfamilies are recognized here: Nippononychidae, Paranonychidae (Paranonychinae Fig. 1, Sclerobuninae Fig. 2), Travuniidae (Briggsinae Fig. 3, Cladonychiinae Fig. 4, Travuniinae Figs 5, 6).

**Figure 7.**
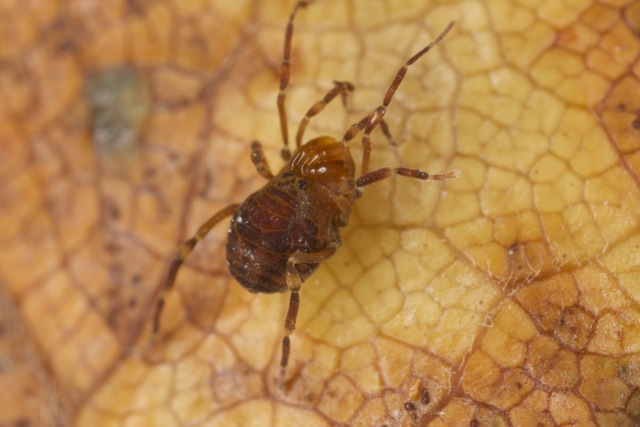
Synthetonychiidae, *Synthetonychia* sp., adult, New Zealand. Photo, ID and copyright © Gonzalo Giribet. Image online at link.

**Figure 8.**
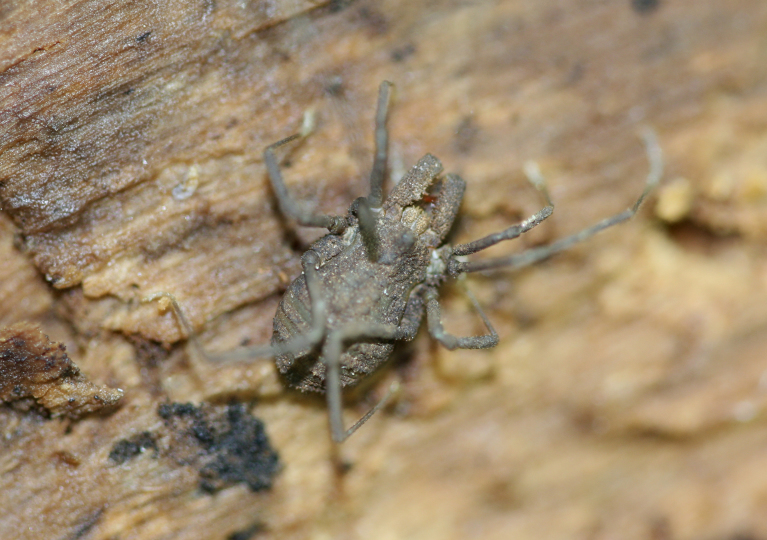
Triaenonychidae, Adaeinae, *Adaeulum* sp. from South Africa, Hogsback. ID by Amanda C. Mendes. Photo and copyright © by Charles Haddad.

**Figure 9.**
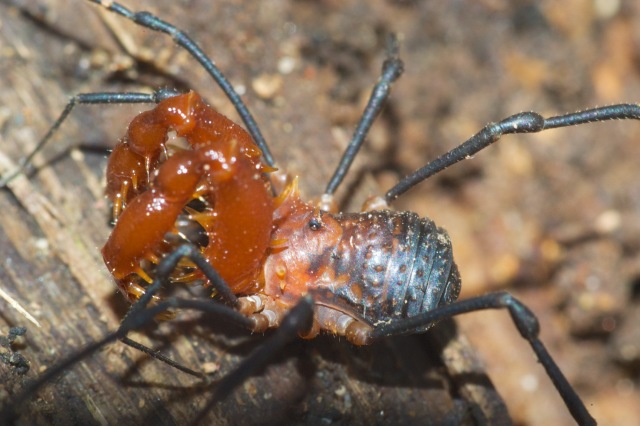
Triaenonychidae, Soerensenellinae, *Soerensenella* sp., adult, New Zealand, Waikato. Photo, ID and copyright © Gonzalo Giribet. Image online at link.

**Figure 10.**
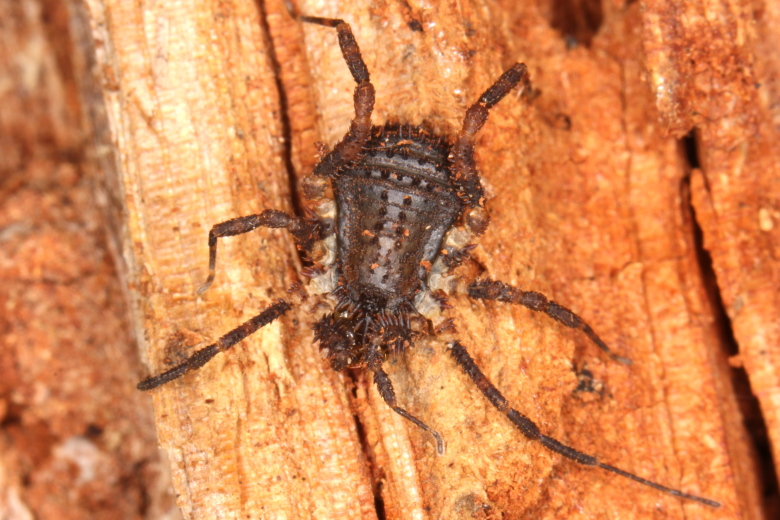
Triaenonychidae, Triaenobuninae, *Triaenobunus* sp. adult male, Australia, Tasmania, Hobart, Tolmans Hill. ID by Adriano B. Kury. Photo and copyright © by Kristi Ellingsen. Image online at link.

**Figure 11.**
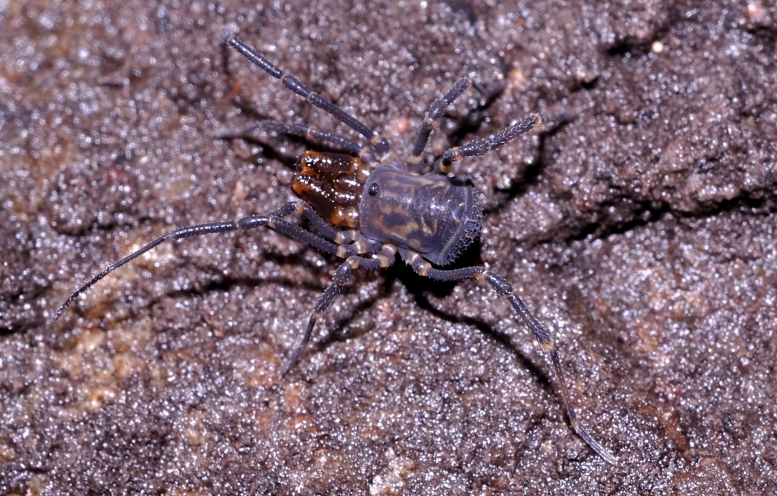
Triaenonychidae, Triaenonychinae, *Ankylonuncia* sp. adult, Australia, Tasmania, Verona Sands, found under rock in boggy ground. ID by Amanda C. Mendes. Photo copyright © by Andrew Bonnitcha. Image online at link.

The Triaenonychoidea include 493 valid species/subspecies, with 307 Australasian, 159 Afrotropical, 26 Neotropical and 1 Nearctic taxa. A single species from the Crozet Islands is listed here as Australasian. Two families and 4 subfamilies are recognized here: Synthetonychiidae Fig. 7, Triaenonychidae (Adaeinae Fig. 8, Soerensenellinae Fig. 9, Triaenobuninae Fig. 10, Triaenonychinae Fig. 11).

### Taxa included

**Table:** 

Rank	Scientific Name	Common Name
kingdom	Animalia	animals
phylum	Arthropoda	arthropods
class	Arachnida	arachnids
order	Opiliones	harvestmen
suborder	Laniatores	
superfamily	Travunioidea	
family	Nippononychidae	
family	Paranonychidae	
subfamily	Paranonychinae	
subfamily	Sclerobuninae	
family	Travuniidae	
subfamily	Briggsinae	
subfamily	Cladonychiinae	
subfamily	Travuniinae	
superfamily	Triaenonychoidea	
family	Triaenonychidae	
subfamily	Adaeinae	
subfamily	Soerensenellinae	
subfamily	Triaenobuninae	
subfamily	Triaenonychinae	
family	Synthetonychiidae	

## Temporal coverage

**Data range:** 1758 1 01 – 2014 8 31.

## Usage rights

### Use license

Open Data Commons Attribution License

## Data resources

### Data package title

World Checklist of Opiliones species (Arachnida). Part 1: Laniatores – Travunioidea and Triaenonychoidea

### Resource link

GBIF: http://ipt.pensoft.net/ipt/resource.do?r=opiliones1

### Number of data sets

1

### Data set 1.

#### Data set name

World Checklist of Opiliones species (Arachnida). Part 1: Laniatores – Travunioidea and Triaenonychoidea

#### Data format

Darwin Core Archive format

#### Number of columns

20

#### Character set

UTF-8

#### Download URL

http://ipt.pensoft.net/ipt/archive.do?r=opiliones1

#### Data format version

1.0

#### Description

**Data set 1.** 

Column label	Column description
taxonID	unique ID for each specie/subspecies
suborder	Laniatores, the only suborder contained in Part 1 of this project
superfamily	name of the superfamily
family	name of the family
subfamily	name of the subfamily
genus	name of the genus
specificEpithet	species name
infraspecificEpithet	subspecies name
scientificNameAuthorship	authority
scientificName	combined full name with author and date
taxonRank	whether it is a species or subspecies
realm	one of the 6 Zoogeographical realms of the world, also Holarctic when combined occurence in Nearctic and Palearctic
taxonomicStatus	if valid or invalid, and in this case only valid names are included
rightsHolder	who detains the copyright
type	it is a checklist
basisOfRecord	it is a dataset
order	not of much use in the context, but important for connection with other bases.
kingdom	not of much use in the context, but important for connection with other bases.
phylum	not of much use in the context, but important for connection with other bases.
class	not of much use in the context, but important for connection with other bases.

## Additional information

### Nomenclatural notes

1. Cladonychiinae originally in Triaenonychidae (Hadži 1935), elevated to family by Cokendolpher (1985) by synonymizing Cladonychiinae with Erebomastridae). Both Cladonychiidae and Briggsidae were fused with Travuniidae by Kury (2013) (See Introduction: Placement and composition). In this paper we downgrade them to subfamilies of Travuniidae: Briggsinae, Cladonychiinae. **New familial assignment.**
2. Forster (1954) synonymized *Nuncia ovata* Roewer, 1915 with *Triaenonyx cockayni* Hogg, 1920, which he considered as a subspecies of *Nuncia coriacea* (Pocock, 1902). But he overlooked the fact that *ovata* is senior to *cockayni* (although junior of *coriacea*) and inverted the precedence. This is corrected here, through the new combination *Nuncia coriacea ovata* Roewer, 1915. Author name should not be within parentheses because it is combined within the same genus, although in a different subspecific arrangement.
3. Forster (1954) changed the name of *Neonuncia enderbei* (Hogg, 1909) to *Neonuncia enderbyi*, better to conform with the spelling of the island name. But this is an incorrect subsequent spelling according to ICZN (32.5.1. – "Incorrect transliteration or latinization, or use of an inappropriate connecting vowel, are not to be considered inadvertent errors."), and the original spelling by Hogg should be conserved.
4. Kury and Mendes (2007) detected that some genera published by Roewer (1935) did not meet ICZN conditions for availability. However, this is not true for *Peltonychia*. Kury & Mendes saw only the heading of this genus, treated by Roewer in page 55, but they overlooked one nomenclatural act buried amidst the introductory text of Roewer, much earlier in the text. On page 12, Roewer explicitly stated: “Damit ist dieses Tier in die Familie der Travuniidae zu verweisen, und wir bezeichnen es mit *Peltonychia leprieuri* (LUCAS) als Genotypus dieser Gattung...” Therefore, *Scotolemon leprieurii* Lucas, 1861 was explicitly designated as type of *Peltonychia* and this genus was already available in Roewer (1935) being the valid senior synonym of *Hadziana*, *contra* Kury and Mendes (2007). As a result, all eight species combined under *Hadziana* are here combined under *Peltonychia*, restoring the combinations used by Martens (1978).
5. ICZN article 13.3 states “To be available, every new genus-group name published after 1930 (except those proposed for collective groups or ichnotaxa) must, in addition to satisfying the provisions of Article 13.1, be accompanied by the fixation of a type species in the original publication [Art. 68] or be expressly proposed as a new replacement name (nomen novum) [Art. 67.8].” Seven generic names published by Lawrence between 1931 and 1933 include more than one species and did not originally have designation of a genus type. All of them only became available in Staręga’s catalogue (Staręga 1992), when he designated a type species for each. They are listed below:

*Larifugella* Staręga, 1992

*Larifugella* Lawrence 1933: 226 [unavailable name, ICZN 13.3].

*Larifugella* Staręga 1992: 279 [type species: *Larifugella afra* Lawrence, 1933, by original designation].

*Austromontia* Staręga, 1992

*Austromontia* Lawrence 1931: 398 [unavailable name, ICZN 13.3].

*Austromontia* Staręga 1992: 282 [type species: *Austromontia silvatica* Lawrence, 1931, by original designation].

*Biacumontia* Staręga, 1992

*Biacumontia* Lawrence 1931: 403 [unavailable name, ICZN 13.3].

*Biacumontia* Staręga 1992: 283 [type species: *Biacumontia paucidens* Lawrence, 1931, by original designation].

*Graemontia* Staręga, 1992

*Graemontia* Lawrence 1931: 413; Kauri 1961: 101; [unavailable name, ICZN 13.3].

*Graemontia* Staręga 1992: 285; Kury 2006: 45 (key to species; distribution map) [type species: *Graemontia bifidens* Lawrence, 1931, by original designation].

*Mensamontia* Staręga, 1992

*Mensamontia* Lawrence 1931: 381 [unavailable name, ICZN 13.3].

*Mensamontia* Staręga 1992: 286 [type species: *Mensamontia morulifera* Lawrence, 1931, by original designation].

*Monomontia* Staręga, 1992

*Monomontia* Lawrence 1931: 416; Lawrence 1933: 222 [unavailable name, ICZN 13.3].

*Monomontia* Staręga 1992: 287 [type species: *Monomontia atra* Lawrence, 1931, by original designation].

*Rostromontia* Staręga, 1992

*Rostromontia* Lawrence 1931: 388; Kauri 1961: 100  [unavailable name, ICZN 13.3].

*Rostromontia* Staręga 1992: 288 [type species: *Rostromontia truncata* Lawrence, 1931, by original designation].

## Supplementary Material

Supplementary material 1 Darwin Core Archive: World Checklist of Opiliones species (Arachnida). Part 1: Laniatores – Travunioidea and Triaenonychoidea Data type: occurences
Brief description: This is a local copy of the same database uploaded to GBIF IPT, but frozen in time.
File: oo_31952.txt AB Kury & DR Souza
